# Breaking Symmetry in Viral Icosahedral Capsids as Seen through the Lenses of X-ray Crystallography and Cryo-Electron Microscopy

**DOI:** 10.3390/v10020067

**Published:** 2018-02-07

**Authors:** Kristin N. Parent, Jason R. Schrad, Gino Cingolani

**Affiliations:** 1 Department of Biochemistry and Molecular Biology, Michigan State University, East Lansing, MI 48824, USA; schradja@msu.edu; 2 Department of Biochemistry and Molecular Biology, Thomas Jefferson University, Philadelphia, PA 19107, USA; 3 Institute of Biomembranes and Bioenergetics, National Research Council, Via Amendola 165/A, 70126 Bari, Italy

**Keywords:** icosahedral symmetry, symmetry mismatch, cryo-EM, cryo-ET, X-ray crystallography, portal protein, bacteriophages, giant viruses, archeal viruses

## Abstract

The majority of viruses on Earth form capsids built by multiple copies of one or more types of a coat protein arranged with 532 symmetry, generating an icosahedral shell. This highly repetitive structure is ideal to closely pack identical protein subunits and to enclose the nucleic acid genomes. However, the icosahedral capsid is not merely a passive cage but undergoes dynamic events to promote packaging, maturation and the transfer of the viral genome into the host. These essential processes are often mediated by proteinaceous complexes that interrupt the shell’s icosahedral symmetry, providing a gateway through the capsid. In this review, we take an inventory of molecular structures observed either internally, or at the 5-fold vertices of icosahedral DNA viruses that infect bacteria, archea and eukaryotes. Taking advantage of the recent revolution in cryo-electron microscopy (cryo-EM) and building upon a wealth of crystallographic structures of individual components, we review the design principles of non-icosahedral structural components that interrupt icosahedral symmetry and discuss how these macromolecules play vital roles in genome packaging, ejection and host receptor-binding.

## 1. Principles of Icosahedral Virus Assembly

The principles governing assembly of icosahedral capsids are well understood and have been reviewed in detail [1,2,3,4,5,6]. A true icosahedral shell displays a combination of 5-fold, 3-fold, and 2-fold symmetry axes, which results in 60-fold redundancy. Assembly of icosahedral viruses with more than 60 copies of the viral capsid protein requires conformational plasticity among individual protein subunits, as they occupy so-called quasi-equivalent positions. In these instances, the viral proteins form two types of oligomeric structures: “hexons” (oligomers of six capsid proteins) that comprise the majority of the capsid facets, as well as “pentons” (oligomers of five capsid proteins) that occupy the 12 5-fold vertices [7]. Capsids with icosahedral symmetry are often described by a “triangulation number”, and the total number of subunits contained within the capsid is a multiple of 60. For example, a *T* = 1 virus has 60 subunits, a *T* = 3 virus has 180 subunits, *T* = 4 has 240 subunits, etc. Icosahedral capsids come in two varieties: spherical, whereby all pentamers are equidistant from the center of the capsid, or prolate, that retains quasi-icosahedral symmetry with one direction of the capsid elongated by additional hexameric rings.

Major structural proteins that build up icosahedral capsids are highly conserved in nature. For example, coat proteins adopt conformations such as the PRD1-like, Bluetongue Virus (BTV)-like, or Hong Kong 97 (HK97)-like folds. These folds are conserved across a wide evolutionary gap extending from phage, eukaryotic and archeal viruses, even though the proteins may share less than ~10% sequence homology [8,9]. Similarly, the scaffolding proteins of double stranded DNA (dsDNA) viruses are generally dimers formed from helix-rich proteins [10,11,12]. Virtually all dsDNA-containing viruses use a scaffolding protein-mediated assembly and utilize a canonical HK97-like coat fold, which was first identified in the crystal structure of the bacteriophage HK97 mature capsid [13]. The HK97-fold is ubiquitous in nature, from bacteriophages, where it was originally discovered, to recently isolated, and distantly related icosahedral tailed viruses that infect halophilic archea [14,15], as well as human pathogens such as *Herpesviridae* [16,17,18]. Furthermore, spherical capsids assembled via the HK97-like fold can adopt a huge variety of *T* numbers, growing larger icosahedral cages to accommodate more complex genomes [19,20,21], which may explain why this coat protein fold is found in many diverse viruses.

In this review, we focus on macromolecular assemblies that interrupt true icosahedral symmetry at a single 5-fold vertex (i.e., the “unique” vertex), generating a symmetry mismatch within the coat shell. Arguably the most common example of symmetry mismatches in icosahedral viruses is the portal protein, which was originally discovered in tailed bacteriophage, although symmetry mismatches are present in viruses that infect all kingdoms of life. Spherical capsids with *T* = 7 symmetry have been enormously well studied in the last two decades, thanks to increased access to better technology within the field of cryo-electron microscopy (cryo-EM), which is ideally suited to image complex molecular machines [22,23,24,25,26]. A wealth of information regarding larger and more complex capsid geometries has also benefitted from current advances in environmental isolation methodology (“phage hunting”) [27,28,29] and access to better laboratory culture schemes, revealing an unprecedented breadth of capsid geometry. Overall, it has become evident that the ability of viral complexes to correctly assemble and function lies within specific conformations that promote relevant protein:protein interactions. Thus, we predict that in analogy to capsid components that obey icosahedral symmetry, which are highly conserved in structure and function, those that break it, are also likely correlated in distantly related families of viruses. This review will explore this hypothesis.

## 2. Asymmetric Cryo-EM Reconstructions Reveal the Portal Protein Occupies a Unique 5-Fold Vertex

**Figure 1:**
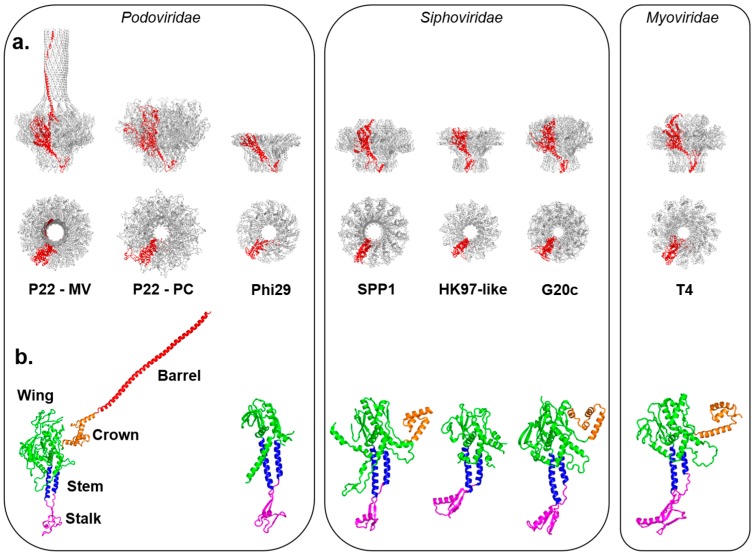
Crystal structures of in vitro assembled portal proteins deposited in the RCSB Protein Data Bank. (**a**) Quaternary structures of portal proteins from *Podoviridae*, *Siphoviridae* and *Myoviridae* shown as side and top views. All structures are aligned with respect to the Mature Virion (MV)-portal protein of P22 and are shown in scale with the protomer “A” colored in red and the rest of the oligomer in gray; (**b**) Tertiary structures of portal protein protomers color-coded to highlight the stalk (magenta), stem (blue), wing (green), crown (orange) and barrel (red). Except for P22 portal protein, which is shown only in the MV-conformation, all other portal protomers are in scale.

**Figure 2:**
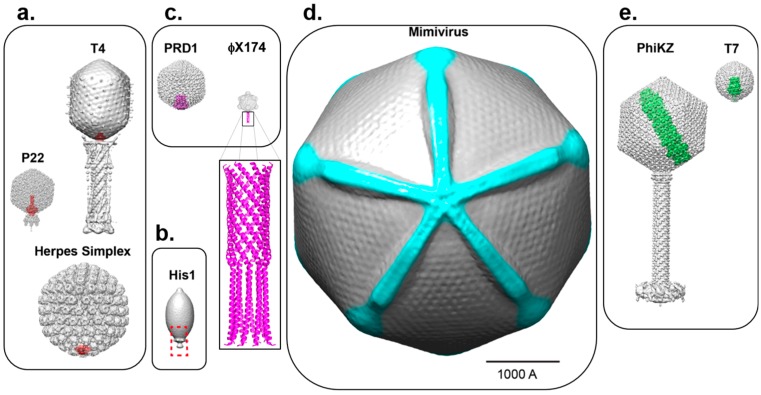
Asymmetric cryo-EM reconstructions of bacterial, archeal and eukaryotic viruses with unique vertices. (**a**) The portal protein (colored in red) occupies of a 5-fold vertex of the icosahedral capsid in P22 (EMD-8005), T4 (EMD-2774) and HSV-1 (EMD-5255); (**b**) The lemon-shaped archeal virus His1 is shown with solely the capsid structure from cryo-EM (EMD-6223) and a red dashed box highlighting the unique vertex; currently no portal or other data are available for this unique assembly site; (**c**) Two examples of viruses with unique vertices (in magenta) induced upon genome ejection: PRD1 (EMD-5984) and ФX174 (EMD-7033). For the latter, the crystallographic structure of the H-protein (PDB 4JPP) is modeled to scale at a 5-fold vertex and is also shown enlarged; (**d**) The Mimivirus capsid (EMD-5039) is rendered with density for the starfish seal highlighted in cyan and is rotated 90° to view down the axis containing the unique vertex; (**e**) The inner body and inner core proteins of phages PhiKZ (EMD-1415, EMD-1996) and T7 (EMD-5568) are shown in green inside their respective capsids. All viruses in Figure 2 are shown in scale (the scale bar is 1000Å).

The portal protein (also known as “head-to-tail connector” in bacteriophages) is one of the best examples of non-icosahedral structures breaking the global symmetry of virus capsids [30]. In tailed bacteriophages and herpesviruses, the portal forms a homo-dodecameric assembly that replaces a penton at a unique 5-fold vertex of the icosahedral capsid [31]. Portal proteins have been investigated using biophysical methods for the last quarter-century, providing an exceptional framework to understand their composition and assembly. All known portals are dodecameric in the context of viral capsids, but can form oligomers of different stoichiometry in vitro [32,33,34,35,36]. This polymorphism reflects oligomerization artifacts obtained in the absence of coat protein, as well as scaffolding protein, which controls the efficiency and rate of portal oligomerization [37]. 3D-structures have been obtained for naïve portal proteins from bacteriophage Ф29 [38,39,40], T7 [41,42], SPP1 [43,44,45], P22 [46,47,48,49], T4 [50], G20c (Protein Data Bank, PDB, entry 4ZJN) and from a prophage of *Corynebacterium diphtheriae* similar to HK97 (PDB 3KDR) (Table 1, Figure 1a). In parallel, cryo-EM single particle analysis of frozen hydrated virions reconstructed without applying icosahedral symmetry (methodology referred to as “asymmetric” reconstructions) enabled direct visualization of the portal vertex in situ [51] (Table 2 and Table 3). In these reconstructions, the density for portal protein is usually noisy, but applying local 12-fold symmetry averaging at the portal vertex improves the signal-to-noise yielding higher local resolution [52,53]. The portal assembly has been visualized in situ in asymmetric cryo-EM reconstructions of Ф29 [54,55], P22 [52,56,57] (Figure 2a), T7 [58], P-SSP7 [59], Syn5 [60], ε15 [61], TW1 [62] as well as phages Sf6 [63] and CUS-3 [64], providing an essential validation of the crystallographic structures solved from in vitro assembled recombinant proteins (Figure 1), and a means to identify new portal proteins in virions, in the absence of other structural information.

The “portal-fold” is highly conserved in nature despite low sequence conservation. With differences in size between ~30 kDa to over 80 kDa (Table 1), the portal-fold is built from four constant regions and one variable region (Figure 1b). The constant regions include a central “wing” that forms a flat domain of α/β-fold; a “stem” formed by two antiparallel α-helices making up most of the DNA channel; a “stalk” that forms the bottom of the portal ring and binds to the large terminase subunit (TerL) as well as tail accessory factors [46]; and a helical “crown” that is flexibly connected to the wing. In all sequenced P22-like *Podoviridae*, the crown extends into a helical ~130-residue long “barrel” [52]. The barrel is not found in phages similar to φ29 [39] and T7 and is less frequently observed in portal proteins from both long-tailed bacteriophage families: *Siphoviridae* and *Myoviridae* [52]. It is believed to help direct ordered packaging of the genome and is required to deliver the DNA and pilot proteins (also known as “ejection” proteins) to the host bacterium efficiently. Surrounded by DNA, the portal barrel folds straight inside the virion, though its structure in situ is slightly twisted as compared to the naïve conformation seen in the crystal structure [65].

Portal proteins are highly plastic molecular assemblies that play a direct role in the packaging reaction [66]. The built-in flexibility of the portal-fold [38] is a key structural determinant that allows the assembly of a dodecameric ring at a 5-fold pentameric vertex. Recent studies revealed the portal protein of phage P22 undergoes a structural maturation during genome packaging [67]. The dodecamer switches from a high affinity state for TerL in the procapsid (referred to as procapsid-portal or PC-portal), which is highly asymmetric (Root-Mean-Square Deviation, RMSD among subunits >2.5 Å), to a symmetric conformation in the mature virion (or MV-portal) [46] that has negligible affinity for the packaging motor. PC-portal asymmetry exposes a quasi-5-fold symmetric surface, which binds TerL with high affinity in vitro [67]. Crystal structures of PC- and MV-portal protein fit accurately in the asymmetric cryo-EM reconstructions of the P22 procapsid [53] and mature virion [52], respectively. However, the quaternary structure of PC-portal is incompatible with packaged DNA coaxially spooled around the portal vertex [67], which led to hypothesize that newly packaged DNA triggers maturation from PC- to MV-conformation and, thus, the portal protein functions as a dynamic sensor that couples icosahedral capsid assembly to genome packaging. Similarly, comparative analysis of different portal protein structures led Guo and collaborators to propose that the portal protein channel acts as a “one-way inward valve” during genome packaging and DNA is packaged by a revolution mechanism [68,69]. Electrophysiological studies of portal proteins from T3, T4, SPP1 and φ29 inserted into lipid bilayers identified a 3-step conformational change in the portal channel during DNA passage, underscoring how the structural plasticity of portal proteins may also aid in DNA ejection [70]. However, the conformational plasticity of portals is not accompanied by macroscopic rotation at the 5-fold vertex during DNA packaging, as revealed by biochemical studies in T4 [71] and single-molecule force spectroscopy with polarization-sensitive single-molecule fluorescence in φ29 [72].

The portal protein also serves as the attachment point for TerL and the tail apparatus. The profound asymmetry and quasi-5-fold arrangement of portal protomers in PC-portal [67] may explain why TerL is often found to bind the portal vertex as a pentamer [73,74,75]. Likewise, the lack of identical binding sites at the interfaces exposed by portal protomers is possibly the reason why head completion proteins that bind at the end of DNA packaging (also known as “head-tail connector”) such as gp4 in P22 [76], gp15 in SPP1 [77] and gpFII in phage λ [78] are monomeric in solution but oligomerize upon binding to the portal vertex. In P22, the symmetry fall-off from the vertex to the tail needle is exemplified by the fact that 12 copies of gp4 assemble onto portal followed by six copies of gp10 [79] and a trimer of the tail needle gp26 [80].

## 3. Cryo-Electron Tomography Identifies a Portal Protein in Herpesviruses

A portal protein was discovered in Herpes Simplex Virus 1 (HSV-1) [81] as gene product pUL6, one of the seven HSV-1-encoded proteins essential for DNA packaging [82]. The portal protein gene is highly conserved in all members of *Herpesviridae* [83] and was also isolated and characterized for Human Cytomegalovirus (HCMV) [84] and Varicella-Zoster Virus (VZV) [85], where it is known as pUL104 and pORF54, respectively. At the structural level, herpesvirus portal proteins are similar in size to large bacteriophage portals (protomer M.W. > 80 kDa) (Table 1). In vitro assembled recombinant portal proteins from HSV-1 [34] and VZV [86] display a funnel-shaped architecture, with features consistent with the crown, wing, and clip domains observed in phage connectors.

Direct observation of herpesvirus portal protein within capsids has been hampered by the small size of this oligomeric assembly, which accounts for less than 5% of the total mass of the coat protein layer, as well as the technical challenge of resolving non-icosahedral components in larger icosahedral viruses (i.e., HSV-1 diameter is ~1250 Å). Cryo-ET of HSV-1 A-capsids (empty, mature capsids) labeled with immuno-gold bound to anti-UL6 antibodies allowed for indirect visualization of the pUL6 at a unique vertex [87]. This study revealed pUL6 is positioned on the outer surface of the capsid floor layer, and unlike bacteriophages, the bulk of its mass lies outside, rather than inside, the capsid floor (Figure 2a). Similarly, cryo-ET of urea-extracted HSV-1 capsids lacking pentons revealed that the dodecameric portal assembly is ~140 Å tall and 110 Å wide and is located partially inside the capsid shell, at a position equivalent to that of phage portals [88]. In a related study, visualization of the portal protein at a unique vertex of the Kaposi’s Sarcoma-Associated Herpesvirus (KSHV) using cryo-ET [89] provided direct structural evidence for the existence of a portal complex in a γ-herpesvirus. Like in HSV-1 (a α-herpesvirus), the portal is localized inside the capsid floor, lacking the external machineries characteristic of portals in bacteriophages.

Using the emerging technique of Zernike phase-contrast cryo-EM, Rochat et al. were able to enhance the image contrast of ice-embedded HSV-1 B-capsids, and generate an image reconstruction of pUL6 in the context of the icosahedral capsid [90]. This work not only provided a higher resolution, more detailed description of pUL6 quaternary structure, but also unequivocally resolved the position of the portal vertex within the B-capsid. The portal is situated beneath one of the pentameric vertices, removing uncertainty from previous, low resolution, cryo-ET studies [87]. More recently, development in cryo-ET image classification using multivariate statistical analysis (MSA)-guided classification and “melon ball” alignment allowed Schmid et al. to image the composition of the unique portal vertex of HSV-1 in intact virions that also contain a lipid membrane and tegument proteins [91]. Strikingly, these authors identified a tail-like assembly specifically at the portal vertex of human HSV-1 virions. This tail-like density, lacking in the reconstruction of HSV-1 B-capsid [90], extends ~550 Å outwards from the portal vertex through the tegument density, connecting the capsid to the virion lipid membrane. This previously unsuspected structure, named Portal Vertex Associated Tegument (PVAT) is functionally similar to a phage tail and its location suggests a role in genome release into the nucleus of the infected host cell. Unlike phage tails, the PVAT is thought to be formed by a set of tegument proteins consistently arranged with respect to the portal vertex as opposed to a static, self-contained structure.

## 4. The Challenge of Visualizing Symmetry Mismatches at the Portal Vertex in Genome Packaging Motors

Icosahedral viruses that contain a portal protein package their genomes through the portal vertex using a genome packaging motor. In tailed bacteriophages, the motor consists of both large and small terminase subunits, referred to as TerL and TerS. Terminase subunits are functionally conserved in *Herpesviridae* that also contain a third subunit (i.e., pUL33 in HSV1 or pUL51 in HCMV), possibly functioning as chaperone or assembly factor [92]. TerL is usually monomeric in solution, and oligomerizes upon binding to the portal vertex. The crystal structures of several phage TerL revealed a bipartite organization built by an N-terminal ATPase domain that contains adenosine triphosphate (ATP)-binding Walker A and B motifs [73,93] flexibly linked to a C-terminal RNAse H-fold nuclease domain [94,95]. These features are also conserved in *Herpesviridae* [96,97]. Much less is known about the functional TerL ring assembled onto portal protein that was visualized using cryo-EM at medium resolution for phage φ29 [75] and at low resolution for phages T4 [73] and T7 [74]. In φ29, a *Bacillus*-phage that also contains a packaging RNA (pRNA) bound to the portal vertex [98], the N-terminal ATPase domain of TerL follows the 5-fold symmetry of the pRNA, which is consistent with the icosahedral 5-fold vertex. In contrast, the X-ray structure of the T4 procapsid bound to TerL revealed five copies of the ATPase associated at the portal vertex. This low resolution structure was interpreted with the N-terminal ATPase domain of TerL docked directly against the portal clip region, projecting the nuclease domains to form a channel [73]. This model disagrees with biochemical studies in phages T4 [99], T3 [100], λ [101] and P22 [102] that found the C-terminus of TerL rather than the N-terminal ATPase domain, contains a dedicated portal protein binding anchor. No structural information on the terminase complex assembled to the portal vertex is available for Herpesviruses.

While TerL appears to oligomerize only upon binding to the portal vertex, the smaller terminase subunit (TerS) is always oligomeric in solution, although the stoichiometry of the TerS oligomerization is not conserved. Nonamers of P22 TerS (gp3) were observed both in solution studies and in crystals [95,103,104,105], and this structure is very similar to the TerS of the *Siphoviridae* SPP1-like phage SF6 [106]. This oligomer is surprisingly different from the octameric TerS of podophage Sf6 [107], and the distant *Myoviridae* T4-like 44RR, which was determined crystallographically as a mixture of *undecamer* and *dodecamers* [108]. In P22, all DNA-binding determinants are confined in a C-terminal basic moiety comprising residues 140–162, which also overlaps with the TerL Binding Domain (LBD) [103]. In contrast, in phage λ TerS (gpNu1) [109] and possibly in Sf6 [110] and T4 [108], DNA-binding is thought to occur via an N-terminal winged helix-turn-helix motif. The way TerS binds to DNA likely varies in different viruses.

TerS and TerL assemble into a pre-packaging complex that is stably populated in solution for λ and P22 as well as HSV-1 [111] but has proven difficult to assemble in vitro for other viruses. The stoichiometry of the pre-packaging complex is consistent with a nonameric TerS bound to two or three copies of TerL in P22. Similarly, in λ the terminase subunits form a stable ~114.2 kDa heterodimer, consisting of one TerL (gpA) bound to two TerS subunits, in equilibrium with a 13.3 S species of ~530 kDa, consisting of four protomers [112,113]. Thus, either the stoichiometry of the pre-initiation complex is different from the packaging motor, or the pentameric state observed in T4 [50,73], T7 [74] and φ29 [75] is not universally conserved in all packaging motors. Further investigation via cryo-EM is essential to clarify the architecture of the packaging motor assembled at the portal vertex during active DNA packaging and under resting conditions.

## 5. Unique Capsid Vertices that Break Icosahedral Symmetry without a Portal Protein

Viruses with asymmetric vertices are prevalent across all kingdoms of life. In addition to truly icosahedral capsids, lemon (Figure 2b) or fullerene shaped capsids are emerging as a common morphology found within hypersaline and hyperthermophyllic environments. Several of these viruses contain a portal-like assembly occupying a single capsid vertex [114,115] and also a tail-like appendage that displays 6-fold symmetry, reminiscent of phage tail spikes. Thus, even in the absence of a true icosahedral capsid, it appears that a 5-fold or 6-fold symmetry mismatch through a portal or ring connector is a common structural motif that extends to *Archea*-infecting viruses. Higher resolution studies are needed to confirm that these structures are indeed analogous to bona fide portal rings, yet these remain challenging owing to the difficulty in cultivating archeal viruses in the laboratory. Despite this feature being apparently universal across kingdoms, some icosahedral viruses entirely lack specialized portals, or analogous ring-like structures, and instead adopt specialized machinery to eject their genomes across biological membranes. These unique features highlight the structural diversity found within virology, and we can predict that additional strategies for genome release are yet to be discovered. Below we focus on a variety of specialized vertices in this category of viruses and highlight a few well-studied examples.

*Microviridae* encompasses some of the smallest icosahedral ssDNA bacteriophages known. ФX174, with a *T* = 1 structure, is a representative member of this group and has been well studied in terms of capsid assembly [116] and structural evolution [117,118,119], as well as the proteins required for host attachment [120,121]. Recently, through a combination of crystallography and cryo-ET (Table 2), the genome delivery apparatus of ФX174 has been revealed. The H “pilot” protein is discreetly packaged inside the capsid until it forms a genome delivery tube at the moment of infection [122,123] (Figure 2c). The mechanism by which ФX174 senses cellular contact and builds this apparatus is not yet known, although functionality of the tube is linked to protein length [124]. Interestingly, the H-protein tube structure is formed by long α-helices and resembles the C-terminal portal protein barrel within *Podoviridae* (Figure 1a and Figure 2c). Thus, even in the absence of a dedicated ring at a predetermined vertex, genome delivery extending to *Microviridae* shares some commonality with tailed phages.

Similarly to ФX174, the membrane-containing dsDNA bacteriophage PRD1 extends a tube from the virion to facilitate genome ejection [125]. This membrane nanotube is formed by the virus-encapsidated internal membrane via two integral membrane proteins, P20 and P22, and extends via a unique vertex (Figure 2c) that is biochemically distinct from the other vertices. These small membrane proteins are necessary for the binding of the putative packaging ATPase P9, via another capsid protein, P6, to the virus particle [126], and appears to localize the proteins involved in genome packaging and facilitate genome ejection at this unique vertex [127]. Furthermore, the integral membrane protein P16 stabilizes the PRD1 adsorption vertex structure [128]. Therefore, although PRD1 requires a membrane for genome delivery, specialized proteins are also needed to coordinate the tube assembly.

On the opposite end of the spectrum to the small *T* = 1 *Microviridae*, are giant viruses (Table 2), which are so large that they can be visualized using a conventional light microscope. Owing to their huge size and complexity (e.g., genomes larger than 1 Mb encoding more than 1000 open reading frames [129,130,131]), the structure and assembly mechanisms of giant viruses have remained poorly understood and difficult to study. One obvious hallmark of giant viruses, and the origin of their moniker, is the presence of large virions that are currently defined to be greater than 300 nm in diameter [132,133,134]. Many of the known giant viruses utilize one or more unique vertices within the capsid to deliver their genomes. In the majority of cases these vertices are sealed by special, likely proteinaceous assemblies (“seal complexes”) that come in two varieties: an external seal that closes a 5-fold “flap”, exemplified by the so-called “starfish vertex” in Mimivirus and Samba virus [135,136,137] (Figure 2d), and an “internal” seal which appears to sit within the capsid, exemplified by the *Pithovirus* and *Mollivirus* cork structures [138,139,140]. For the cork-containing giant viruses, it is unclear how these capsids are assembled, as initial microscopy studies do not reveal evidence that they are formed by regular capsid lattices. By contrast, icosahedral giant viruses such as Mimivirus and Samba virus likely assemble according to pentasymmetron and trisymmetron organization, which are large arrangements of capsomers centered around 5-fold and 3-fold symmetry axes [141], respectively, although the rigidity and homogeneity of these two viruses differ significantly [133,142,143]. In addition to the starfish seal, an asymmetric, internal feature of the giant viruses is a membrane bound nucleocapsid that aids in genome release [144,145]. Following genome release, a lipid membrane sac of unknown structure or function is left inside the Mimivirus and Samba virus capsids [133,135]. It is currently unclear if this plays a role in genome release or assembly. It remains to be determined if all, or most icosahedral giant viruses assemble with a similar capsid organization or utilize similar vertex flaps. Very limited information is currently available for giant viruses at the mechanistic level, and no information is currently present at the atomic level. However, technological advances in cryo-ET and single particle cryo-EM are breaking down barriers almost daily. These are soon to be applied to the study of giant viruses, as just recently the newest record for size has been extended to cryo studies of a *Pithoviridae* giant that is 2.5 μm in length and 0.9 μm in diameter [146,147].

## 6. Bubblegrams and Cryo-ET Reveal Asymmetrically Organized Proteins inside Icosahedral Capsids

Bubblegram imaging involves repeatedly exposing a vitrified specimen to the electron beam until the sample builds up radiation damage. This damage releases small bubbles of hydrogen gas, which in all published reports to date have been used to identify the composition and the location of a proteinaceous component surrounded by a genome within a virus particle. This technique is gaining traction as a means to characterize novel capsid structures, and has been particularly useful to visualize phage proteinaceous assemblies that are surrounded by dsDNA genomes. This technology was pioneered to study the *Pseudomonas* phage ϕKZ, a large *T* = 27 phage with a highly organized internal nucleocapsid termed the “inner body” [148]. Cryo-EM studies were able to reconstruct entire ϕKZ virions, and suggested a role for the inner body during DNA packaging [149] (Figure 2e). Solving the three dimensional structure of the inner body required a combination of bubblegrams, to locate and orient the inner body within the ϕKZ capsids, and difference maps [150]. This combination of methods also allowed characterization of the inner body composition and positioning in other *Pseudomonas* giant phages, EL and Lin68 [151].

DNA bacteriophages T7 [58], ε15 [61] and P-SSP7 [59] possess an internal core of ejection proteins located above the portal protein crown, which play a vital role in the delivery of their genomes across the cell envelope of Gram-negative bacteria. In T7 and Syn5, the “core” consists of three proteins, gp14/gp15/gp16 (Figure 2e) [36,152] and gp43/gp44/gp45 [153], respectively, but the core is replaced by a single polypeptide chain in ε15 (gp17) [61] and possibly P-SSP7 [59]. The density for ejection proteins, which are concentrically arranged above the portal vertex, is often very noisy in traditional asymmetric cryo-EM reconstructions of the mature virions. Computational algorithms such as “focused asymmetric reconstructions” were then used to achieve higher resolution by eliminating smearing from “combinatorial assembly isomerism” or variations of core proteins stacking between particles [154]. It appears that T7 contains 12 copies of gp14, eight copies of gp15 and only four copies of gp16. Likewise, bubblegrams were also useful to map individual proteins that comprise the T7 “core” [155], yet these reconstructions were limited in resolution. Core proteins undergo dramatic structural rearrangements upon genome ejection, which triggers internal core protein disassembly and DNA release [58,156]. Cryo-ET and subtomogram averaging have been instrumental to study the interactions between host cells and phages in the act of ejecting DNA. Mounting evidence suggests the core proteins are ejected inside the bacterial envelope just prior to DNA to form long extensible channels that protect viral DNA during transport into the host. Tubular features consistent with a DNA-channel were observed using cryo-ET for ε15 [156], T7 [157] as well as cyanophages Syn5 [60] and P-SSP7 [158] during infection of *Salmonella*, *Escherichia coli* and *Prochlorococcus marinus* cells, respectively.

Internal proteins are also present in phages similar to P22, where they lack the ordered arrangement above the portal vertex seen in T7. The ejection-protein (“E-proteins”) gp7, gp16 and gp20 are ejected into the host along with the P22 genome and are proposed to protect the DNA in the bacterial periplasm during infection. Like T7, the ejection proteins in P22 are present in the capsid in different copy numbers: 12 copies of gp7 and gp16, but unlike T7, there are as many as 30 copies of gp20 [159]. Gp16 acts within the first 10 min of infection, and when gp16-deficient phage are present, cells continue to divide normally and do not replicate any phage genome [160,161]. Co-infections showed that gp16 can work in trans, complementing a gp16-deficient particle, indicating that its essential early function occurs once DNA is ejected inside the host. Furthermore, gp16 deficient phage do not induce the superinfection exclusion response and gp16 is not part of the replication machinery [162]. Indirect evidence supports a membrane-breaching role, as purified gp16 disrupts dye-loaded, lipid vesicles [163]. Recently, biophysical characterization of genome ejections in vitro demonstrates that these E-proteins are released prior to genome exit [164]. These proteins must be first packaged into procapsids during assembly, undergo the maturation event as the dsDNA genome is packaged, and then ultimately be released through the portal protein complex to interact with a membrane bilayer in order to ensure a successful infection. None of the three E-proteins had been revealed through traditional single particle reconstructions despite high resolution, asymmetric [52,53] and symmetrized [165] image reconstructions of mature virions. In the case of P22, bubblegrams revealed that gp7, gp16 and gp20 associate loosely around the portal protein [159] in mature virions. The location and distribution of these proteins in precursor procapsids is unknown, and remains an area open for investigation.

The loose association of the P22 E-proteins around the portal is unlike the highly organized structures of the ϕKZ inner body or the T7 and ε15 cores (Figure 2e). The ϕKZ inner body has been implicated as having crucial roles in DNA packaging and ejection, and remains inside the capsid after infection. In T7, the core has dual requirements: maintaining structural rigidity to facilitate DNA spooling [166,167] but also possessing enough plasticity to undergo extensive rearrangements during DNA ejection into the host as visualized by cryo-ET [157]. In P22, the E-proteins are known to be important for infection, yet no obvious defects in maturation or DNA packaging are observed when these proteins are absent, eliminating a need for these proteins during DNA spooling. Perhaps the degree of rigidity of E-proteins within phage capsids is correlated to the different functional roles these proteins modulate during genome ejection.

## 7. Trimeric Fiber-Penton Base Interaction in the Adenovirus Lineage

**Figure 3:**
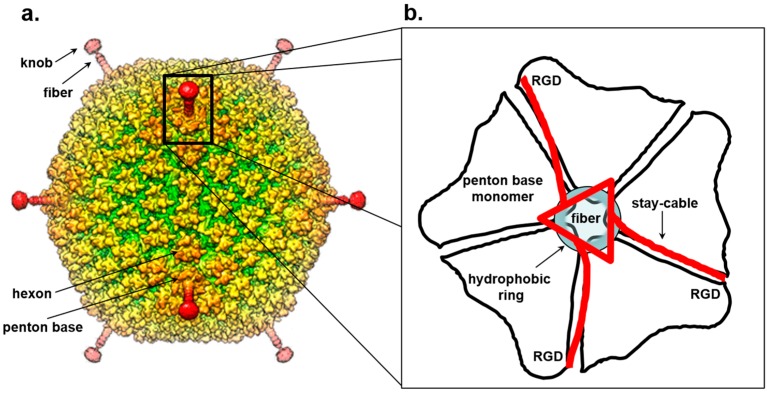
Symmetry mismatch between trimeric fiber and pentameric penton base of adenovirus. (**a**) Surface rendered view of 3D-dimensional reconstruction of human adenovirus 26 (EMD-8471). (**b**) Schematic model for the attachment of adenovirus trimeric fiber to the penton base. The fiber is drawn as a red triangle with extended stay-cables interacting asymmetrically with the pentameric penton base (in black). The schematic diagram is drawn as proposed by Hong Zhou and collaborators in [178]. Hydrophobic residues in the penton base located at the rim of the channel are shown as a blue circle.

Symmetry mismatches in icosahedral DNA viruses also play a critical role in mediating cell adsorption and binding to cell surface receptors. Perhaps the best characterized example in this regard is found in Adenovirus [168] and the evolutionarily-related bacteriophage PRD1. Adenovirus has a pseudo *T* = 25 icosahedral capsid built by 720 copies of a major capsid protein organized as 240 hexon trimers and 60 copies of base subunits organized as pentamers, each occupying the 12 icosahedral vertices. Both X-ray [169] and cryo-EM [170,171] structures of the entire Adenovirus capsid were recently reported (Figure 3a). The penton forms a base [172] that provides an attachment point for flexible trimeric fibers involved in receptor attachment [173] and is directly involved in contacting cell surface integrins [174]. Each penton monomeric subunit has a bipartite structure built by a jelly roll-domain facing the capsid interior and an upper, solvent-exposed insertion domain that binds the N-terminal domain of each fiber. The fiber folds into a homo-trimeric protein consisting of three domains [175]: an N-terminal attachment domain that associates with the penton, a central shaft of variable length formed by a repeating β-spiral motif and a C-terminal head domain involved in receptor-binding [176].

The association of the fiber 3-fold N-terminal domain with the penton base generates an obvious symmetry mismatch that has attracted numerous investigations. A peptide spanning the fiber 21 N-terminal residues of adenovirus 2 co-crystallized with the penton base revealed strong electron density for a central 11-residue portion of the fiber tail peptide spanning residues 10–20 that includes a FNPVYPY motif, which is highly conserved among diverse adenovirus serotypes [172]. This crystal structure, failed to decipher the symmetry mismatch between penton and fiber. Five copies of the fiber peptide were observed in a groove exposed on the outer surface of the penton, formed by adjacent monomers. Likewise, visualization of the fiber density in the cryo-EM reconstruction of the complete virion structure is complicated by both the intrinsic fiber flexibility and the icosahedral symmetry imposed during image reconstruction that results in blurred density for all five copies of the fiber N-terminal moiety [177]. In the 3.6 Å cryo-EM structure of adenovirus [170,178], residues 7–19 of the fiber base previously seen in the crystal structure [172] could be traced revealing three flexible N-terminal tails inserted into three of the five grooves formed by neighboring subunits of the penton base (Figure 3b). This suggests a model for fiber attachment to the penton base whereby the fiber shaft interacts with a hydrophobic ring located at the rim of a channel located inside the penton base, while the N-terminal fiber tails (referred to as “stay-cables”) bind to the grooves between two adjacent penton base monomers making contacts with an Arginine-Glycine-Aspartic acid (RGD) motif in the penton base (Figure 3b). This arrangement not only provides flexibility to the fiber so that it can readily dissociate from the virion upon entry but also overcomes the symmetry mismatch with the penton base by forming a metastable interaction between “stay-cables” and the top of the penton base. Interestingly, the RGD motif is also an integrin binding-site [179,180] suggesting a possible mechanism for fiber dissociation upon integrin-mediated entry into a host cell.

Unlike the cryo-EM density map, in the crystal structure of Adenovirus 35F [169], the central channel generated by five penton copies is almost double the diameter (50 versus 28 Å) than in the isolated penton base [172]. The penton central channel is filled by a strong electron density along the icosahedral 5-fold symmetry axes that was interpreted as a fiber shaft ~90 Å in length corresponding to 5–6 β-spiral. The ability of a fiber shaft to retract inside the penton, inserting into the center of the pentamer, suggests structural flexibility at the penton base whereby the symmetry mismatch between a 5-fold vertex and 3-fold fiber may allow the penton:fiber complex to undergo mutual conformational changes. While it is possible this dynamicity was triggered in the crystal by the presence of calcium ions present in the crystallization buffer [169,174], conformational changes in the penton base upon fiber and integrin binding have also been observed by cryo-EM [181,182], suggesting the penton is intrinsically plastic.

Unlike Adenovirus, PRD1 contains two vertex proteins, the spike protein P5 and the receptor binding protein P2 [183]. P5, a trimeric fiber structurally similar to the adenovirus fiber, contains a fibrous shaft and a C-terminal knob domain [184] that is structurally similar to the tail needle knob of bacteriophage Sf6 [63,185]. P5 attaches to the center of the pentameric penton base protein P31 generating a similar symmetry mismatch as described above for Adenovirus. Interestingly, P31 and P5 share high sequence identity at their N-terminal ends that led to the interesting possibility the two proteins form a mixed hetero-pentamer that removes potential symmetry mismatch between the pentameric P31 and the trimeric P5 [186]. While there is no direct evidence in support of this hypothesis, the icosahedrally averaged crystal structure of PRD1 has no electron density for the N-terminal attachment domain of P5 bound to the pentameric P31 [187], supporting the idea of a symmetry mismatch between the two proteins. Unlike the trimeric P5, the receptor binding protein P2 is a monomer that folds into an elongated seahorse-shaped molecule containing a β-propeller “head” displaying pseudo-6-fold symmetry and a long “tail” [186]. P5 and P2 form two separate spikes, interacting with each other near the capsid shell [188]. Direct visualization by cryo-EM revealed the two spikes can adopt several conformations, likely by virtue of their flexibility. While P5 emanates from the center of the penton protein P31, the exact sites of interaction between P2, P5 and P31 are unknown. The symmetry mismatch among the three vertex proteins P2 (a monomer), P5 (a trimer) and P31 (a pentamer) remains unresolved and difficult to study using current structural methods. The presence of two fibers at one penton is not unique to PRD1, but shared by certain avian stains of Adenovirus, which are thought to use two fibers to bind to two different host receptors [189].

## 8. Conclusive Remarks: The Evolution of Structural Methods to “Observe” Asymmetric Features in Icosahedral Capsids

Forty years after the first crystal structure of an icosahedral virus [190] and over three decades since the first cryo-EM reconstruction of Sindbis virus was reported [191], it has become clear that icosahedral viruses are extraordinarily more complex and dynamic than the apparently rigid, 60-fold symmetric icosahedral “cage” would suggest. Proteinaceous assemblies that break the icosahedral symmetry exist in viruses that infect all kingdoms of life, with a remarkably multi-faceted array of topologies and folds. As illustrated in this review, symmetry mismatches in icosahedral DNA viruses not only provide a convenient solution to the problem of packaging and ejecting genetic material, but also play a critical role in the processes that mediate cell adsorption and binding to cell surface receptors.

The inherent challenge of studying non-icosahedral features using traditional cryo-EM experiments and image reconstruction methods is related to being able to locate small proteins or protein assemblies nestled in the inner volume of large viral capsids. If these proteins are not well ordered, the signal can be lost due to the inherent averaging procedures common in many image reconstruction schemes [192,193]. Furthermore, the strong electron density stemming from the highly pressurized and tightly packaged dsDNA genomes may shield the signal for these proteins. With the rapidly expanding possibilities of cryo-EM, such as better instruments, advances in direct detector cameras, and more advanced software, the study of asymmetric features in icosahedral capsids is expected to gain greater and greater popularity. In all cases, the incorporation of phase plate technology and “focused asymmetric reconstruction” algorithms have proven useful to visualize small features that disobey icosahedral symmetry. Importantly, even under ideal experimental conditions, most asymmetric features revealed using cryo-EM methods are visualized at medium resolution (~10 Å). Thus, the availability of high resolution structural models solved in isolation using classical crystallographic and NMR methods is essential to make sense of these EM densities, at least until further improvements in cryo-EM methodology becomes more commonplace.

Finally, one area of research that requires innovation for advancement is in regards to difficult to cultivate viruses, and viruses that are difficult to study genetically. For example, thermophilic and halophilic viruses have challenging growth conditions for laboratory settings, and giant viruses have enormously complex genomes. Both archeal and giant viruses have few genetic tools currently available. Additional methodologies are clearly needed if we are to achieve an atomic or mechanistic level understanding of these systems, even as cryo-EM imaging methods continue to be developed.

As we move forward and continue to uncover the breadth of viral structures, data availability and archiving are becoming increasingly important. Access to the Protein Data Bank (“PDB”) enables structural comparisons such as those described for the portal complexes above. Although the use of the Electron Microscopy Data Bank (“EMDB”) is becoming more widespread, further improvements to universal publication and dissemination policies are needed to fully harness the power of structural virology and marry the power of X-ray crystallography and cryo-EM.

## Figures and Tables

**Table 1:** Inventory of 3D structures of portal proteins deposited in the Electron Microscopy Data Bank (EMDB) and PDB databases.

	Virus	Protomer M.W. (kDa)	Accession Number(s)		Cryo-EM	X-ray
			EMDB	PDB		
*Bacteriophages*	P22 (MV)	82.7	5049, 5050, 5051, 1482, 1483	5JJ3, 4V4K	+++	+++
	P22 (PC)	82.7	5375 *	5JJ1	+++	+++
	Phi29	35.9		1IJG, 1JNB, 1H5W, 1FOU		+++
	T7	59.1	1231, 2356, 2717, 5690	3J4A	+++	
	SPP1	57.3	2993, 2994 1021	2JES	+++	+++
	HK97-like	32.9		3KDR		+++
	G20C	49.7		4ZJN		+++
	T4	61.0	6324	3JA7	+++	
	p2	39.1	2463		+++	
*Herpes*	HSV-1	74.2	5260 ** 5261 ***		+++	

“+++” indicates the technique used to solve the structure; * Visualized in situ (EMD-1827) and symmetrized; ** Visualized in situ (EMD-5255) and symmetrized; *** Visualized in situ (EMD-5255) and unsymmetrized.

**Table 2:** Inventory of asymmetric cryo-EM and cryo-electron tomography (cryo-ET) structures of DNA bacteriophages that contain protein complexes that break icosahedral symmetry.

	Virus	EMDB Accession Number(s)	Cryo-EM	Cryo-ET	Year
*Podoviridae*	Phi29	1506, 5010	+++		2009
	Phi29	1419, 1420	+++		2008
	Phi29	6560	+++		2016
	CUS-3	5946	+++		2014
	Sf6	5730	+++		2013
	T7	5566–5573	+++		2013
	T7	5534–5537		+++	2013
	C1	5446	+++		2012
	P22	1119	+++		2005
	P22	1220	+++		2006
	P22	1222	+++		2006
	P22	1827	+++		2011
	P22	5348, 5231	+++		2011
	P22	8258–6261	+++		2016
	P22	8005	+++		2016
	P-SSP7	1707		+++	2010
	P-SSP7	6427		+++	2016
	P-SSP7	1714, 1715	+++		2010
	P-SSP7	3131		+++	*TBP*
	Syn5	5743–5746		+++	2013
	ε15	1175	+++		2005
	ε15	5203, 5204		+++	2010
	ε15	5207–5209	+++		2010
	ε15	5216–5219		+++	2010
	BPP-1	1619		+++	2010
	N4	1475	+++		2009
	PRD-1	3548–3550		+++	2017
	PRD-1	2438–2440		+++	2013
	K1E	1336	+++		2007
	K1-5	1337	+++		2007
*Myo*	PhiKZ	1415	+++		2007
	T4	1572, 1573	+++		2008
	T4	6323	+++		2015
	T4	2774, 6078–6083		+++	2015
*Sipho*	P2	2463, 2464	+++		2013
	Araucaria	2335–2338	+++		2013
	1358	2820	+++		2016
	TW1	7070, 8854, 8867, 8868	+++		2017
ssDNA	ΦX174	7033, 8862	+++		2017

“+++” indicates the EM technique used to solve the structure.

**Table 3:** Inventory of asymmetric cryo-EM/cryo-ET structures of eukaryotic and archeal viruses with DNA genomes that contain protein complexes that break icosahedral symmetry.

	Virus	EMDB Accession Number(s)	Cryo-EM	Cryo-ET	Year
Archeal	APBV1 *	3857–3859	+++		2017
	His1 *	6220–6222		+++	2015
Eukaryotic	HSV-1	5452, 5453		+++	2012
	HSV-1	5255, 5260, 5261	+++		2011
	HSV-1	1305–1308		+++	2007
	KSHV	1320		+++	2007
	Faustovirus	8144, 8145	+++		2016
	PBCV-1	1597	+++		2009
	PBCV-1	5384	+++		2012
	CroV **	8748	+++		2017
	Mimivirus **	5039	+++		2009
	Samba virus **	8599		+++	2017
	Chilo Iridescent Virus **	1580	+++		2009

“+++” indicates the EM technique used to solve the structure; * Non-icosahedral virus; ** Giant Viruses.
